# Supplementary material for: The human thalamus orchestrates neocortical oscillations during NREM sleep
Source: Nat Commun. 2022 Sep 5;13:5231. doi: 10.1038/s41467-022-32840-w (PMC9445182; doi:10.1038/s41467-022-32840-w)
Supplement: Supplementary file 1 — Supplementary Information [file 41467_2022_32840_MOESM1_ESM.pdf]

Supplementary Information for:

The human thalamus orchestrates neocortical oscillations during NREM sleep.

Thomas Schreiner[1], Elisabeth Kaufmann[2], Soheyl Noachtar[2], Jan-Hinnerk Mehrkens[3] & Tobias Staudigl[1]

1 Department of Psychology, Ludwig-Maximilians-Universität München, Munich, Germany

2 Epilepsy Center, Department of Neurology, Ludwig-Maximilians-Universität München, Munich, Germany

3 Department of Neurosurgery, Ludwig-Maximilians-Universität München, Munich, Germany

Supplementary tables

Supplementary Table 1 | Sleep architecture: Data are means ± s.e.m. N1, N2: NREM sleep stages N1 & N2, SWS: slow-wave sleep, REM: rapid eye movement sleep, WASO: wake after sleep onset. TST: total sleep time (in minutes).

	N1	N2	SWS	REM	WASO	TST [min]
Sleep stage [%]	6.1 ± 1.7	47.4 ± 4.2	16.5 ± 3.5	12.7 ± 2.2	15.9 ± 3.5	529.4 ± 30.1

Supplementary Table 2 | scalp electrodes: overview of scalp electrode that were selected for the respective analyses.

	ANT-SO	ANT-spindle	MD-SO	MD-spindle
P1	Fz	Fp1	Fz	P4
P2	F4	F4	FP2	Pz
P3	F8	F4	F4	F4
P4	FT10	Pz	FT10	C3
P5	F4	Pz	F3	P3
P6	Fz	C3	Fz	Pz
P7	FT10	C4	Fz	Pz
P8	-	-	F7	Cz

Supplementary Table 3 | Sleep Oscillations ANT: Data are means ± s.e.m. Number of detected events and percentage of paired events (i.e., percentage of co-occurring events across sites, relative to the overall number of events at given site) in NREM sleep at ANT contacts and scalp electrodes. Density (events/min of NREM sleep). Two-sided dependent-samples t-test were used, without correcting for multiple comparisons.

	ANT	Scalp	t	*p*
SO number	1513.5 ± 130.1	1573.8 ± 129.9	1.11	0.29
SO density	5.87 ± 0.24	6.29 ± 0.21	1.32	0.21
SO-coupling [%]	33.74 ± 3.02	31.11 ± 3.25	2.07	0.06
spindle number	2203.8 ± 213.5	1903.3 ± 211.5	3.19	0.008
spindle density	9.03 ± 0.65	6.09 ± 1.08	2.56	0.017
spindle-coupling [%]	43.8 ± 4.2	37.3 ± 4.1	3.43	0.005
SO-spindle number	431 ± 52.1	304.1 ± 69.8	3.18	0.008

Supplementary Table 4 | Sleep Oscillations MD: Data are means ± s.e.m. Number of detected events and percentage of paired events (i.e., percentage of co-occurring events across sites, relative to the overall number of events at given site) in NREM sleep at MD contacts and scalp electrodes. Density (events/min of NREM sleep). Two-sided dependent-samples t-test were used, without correcting for multiple comparisons.

	MD	Scalp	t	*p*
SO number	1453.9 ± 121.3	1543.4 ± 114.9	1.06	0.31
SO density	6.21 ± 0.22	6.66 ± 0.22	-0.5	0.61
SO-coupling [%]	32.81 ± 2.31	31.16 ± 3.15	1.37	0.35
spindle number	2093.1 ± 229.3	1464 ± 252.6	3.35	0.05
spindle density	9.18 ± 0.98	5.11 ± 0.91	3.14	0.004
spindle-coupling [%]	29.6 ± 3.7	45.1 ± 5.5	2.3	0.027
SO-spindle number	488 ± 53.3	338.5 ± 79.7	1.78	0.095

Supplementary Table 5 | SO features: Data are means ± s.e.m. Duration and relative occurrence during N2 and N3 sleep for scalp (ANT and MD analyses related), ANT and MD derived SOs.

SO	Duration [sec]	Slope [µV/sec]	Rel. N2 [%]	Rel. N3 [%]
scalp $_{ANT}$	1.31 ± 0.02	67.55 ± 6.63	47.18 ± 4.93	52.81 ± 4.93
scalp $_{MD}$	1.35 ± 0.02	86.19 ±9.81	49.21 ± 4.38	50.78 ± 4.38
ANT	1.31 ± 0.01	75.99 ± 9.61	46.56 ± 3.87	53.43 ± 3.87
MD	1.30 ± 0.02	63.10 ± 4.65	47.70 ± 4.89	52.29± 4.89

Supplementary Table 6 | spindle features: Data are means ± s.e.m. Peak frequency, duration and relative occurrence during N2 and N3 sleep for scalp (ANT and MD analyses related), ANT and MD derived spindles

spindles	Peak-Freq [Hz]	Duration [ms]	Rel. N2 [%]	Rel. N3 [%]
scalp $_{ANT}$	12.97 ± 0.09	0.76 ± 0.03	70.66 ± 4.95	29.33 ± 4.95
scalp $_{MD}$	13.14 ± 0.13	0.77 ± 0.05	70.75 ± 4.61	29.24 ± 4.61
ANT	13.17 ± 0.09	0.80 ± 0.03	73.51 ± 3.76	26.49 ± 3.76
MD	13.10 ± 0.08	0.74 ± 0.03	73.11 ± 3.77	26.88 ± 3.77

Supplementary Table 7 | drug regimen at the time of recordings.

	Anticonvulsant
P1	Levetiracetam, Phenytoin, Lamotrigine
P2	Levetiracetam, Lamotrigine
P3	Lacosamide, Oxcarbazepine, Topiramate
P4	Levetiracetam, Lamotrigine, Oxcarbazepin, Zonisamide
P5	Levetiracetam, Lamotrigine
P6	Lamotrigine, Lacosamide, Zonisamide
P7	Oxcarbazepin, Topiramate
P8	Topiramate

Supplementary Table 8 | Documented effects drugs on sleep architecture.

Drug	Effect on sleep
Lacosamide[1]	none
Lamotrigine[2]	REM↑, N2↑, SWS↓
Levetiracetam[3]	N2↑, REM↓
Oxcarbazepine	unknown
Phenytoin[4]	SWS↓, REM↓
Topiramate[5]	none
Zonisamide[6]	none

1) Hudson J.D., Guptil J.T., Bynes W., et al. (2015). Assessment of the effects of lacosamide on sleep parameters in healthy subjects. Seizure; 25: 155-159 2) Placidi F, Marciani MG, Diomedi M, et al (2000). Effects of lamotrigine on nocturnal sleep, daytime somnolence and cognitive functions in focal epilepsy. Acta Neurol Scand;102:81–86. 3) Cicolin A, Magliola U, Giordano A, et al. (2006). Effects of levetiracetam on nocturnal sleep and daytime vigilance in healthy volunteers. Epilepsia; 47:82–85. 4) Benjamin Legros & Carl W Bazil (2003). Effects of antiepileptic drugs on sleep architecture: a pilot study. Sleep Med; 4(1):51-5. 5) Bonanni E, Galli R, Maestri M, et al. (2004). Daytime sleepiness in epilepsy patients receiving topiramate monotherapy. Epilepsia; 45:333–337. 6) Romigi A, Izzi F, Placidi F, et al (2013). Effects of zonisamide as add-on therapy on sleep-wake cycle in focal epilepsy: a polysomnographic study. Epilepsy Behav; 26:170–174.

Supplementary Table 9 | **Descriptives Slow and Fast spindles:** Data are means ± s.e.m. Number of detected events and percentage of paired events between thalamic contacts and scalp electrodes. Density (events/min). Two-sided dependent-samples t-test were used, without correcting for multiple comparisons.

ANT spindle	slow	fast	t	*P*
spindle number	1916 ± 174.4	1856.5 ± 199.6	0.1	0.65
spindle density	7.87 ± 0.41	7.59 ± 0.71	0.4	0.67
spindle-coupling [%]	26.1 ± 3.8	30.2 ± 5.1	-0.7	0.47
Peak Freq. Hz	10.29 ± 0.15	14.02 ± 0.11		
MD spindle				
spindle number	1920.9 ± 158.6	1767.5± 188.6	1.3	0.20
spindle density	8.31 ± 0.49	7.84 ± 0.87	1.2	0.37
spindle-coupling [%]	24.5 ± 2.7	25.6 ± 4.5	-0.2	0.79
Peak Freq. Hz	10.20 ± 0.12	14.08 ± 0.12		

Supplementary figures

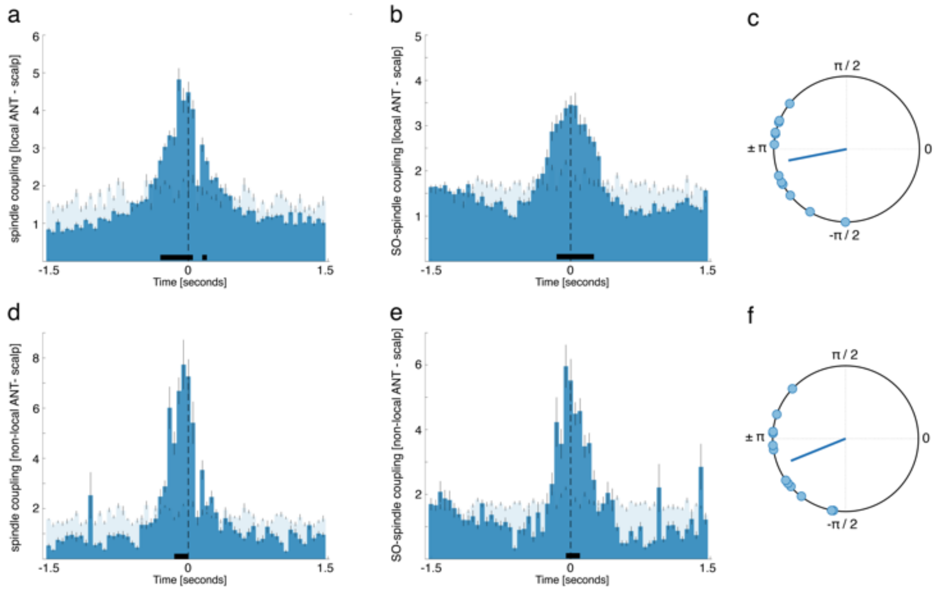

Supplementary Fig. 1 | Local and non-local ANT spindles. Non-local ANT spindles were defined as spindles identified in the ANT whose peak (maximally negative amplitude) overlapped with MD spindle peaks ± 50 ms (267.91 ± 51.57 ANT spindles out of 2203.8 ± 213.5; 11.78 ± 1.84%). All other spindles identified in the ANT were defined as local ANT spindles. The same temporal dynamics with regards to thalamo-cortical spindle coupling and SO-spindle coupling were found for local and non-local ANT spindles. **(a)** Occurrence probabilities of local ANT spindle peaks relative to neocortical spindle peaks (maximal negative amplitude, time = 0; dashed line; bin size = 50 ms), indicating that local ANT spindles precede neocortical spindles (dependent-samples t-test, two-sided, positive clusters from -0.3 to -0.05 sec, $p < 0.0001$ & 0.2 to 0.25 sec, $p = 0.023$; corrected for multiple comparisons across time; time of peak: -0.1 sec). **(b)** Local ANT spindles preferential emerged around ANT SO down states (dependent-samples t-test, two-sided; -0.15 to 0.25 sec; $p = 0.009$; corrected for multiple comparisons across time; peak: 0 sec). **(c)** Phases of the SO-spindle modulation derived from local ANT events. Local ANT spindles started specifically around the ANT SO down states (mean coupling direction: −168.93° ± 10.10°; Rayleigh test, one-sided: $z = 7.93$, $p < 0.0001$). **(d)** Occurrence probabilities of non-local ANT spindle peaks relative to neocortical spindle peaks, indicating that non-local ANT spindles likewise precede neocortical spindles (dependent-samples t-test, two-sided; positive cluster from -0.15 to 0 sec, $p < 0.017$; corrected for multiple comparisons across time; time of peak: -0.05 sec). **(e)** Non-local ANT spindles also preferential emerged around ANT SO down states (dependent-samples t-test, two-sided; -0.05 to 0.1 sec; $p = 0.017$; corrected for multiple comparisons across time; peak: -0.05 sec). **(f)** Phases of the SO-spindle modulation derived from non-local ANT events. Non-local ANT spindles started specifically around the ANT SO down states (mean coupling direction: −157.91° ± 10.21°; Rayleigh test, one-sided: $z = 7.86$, $p < 0.0001$).

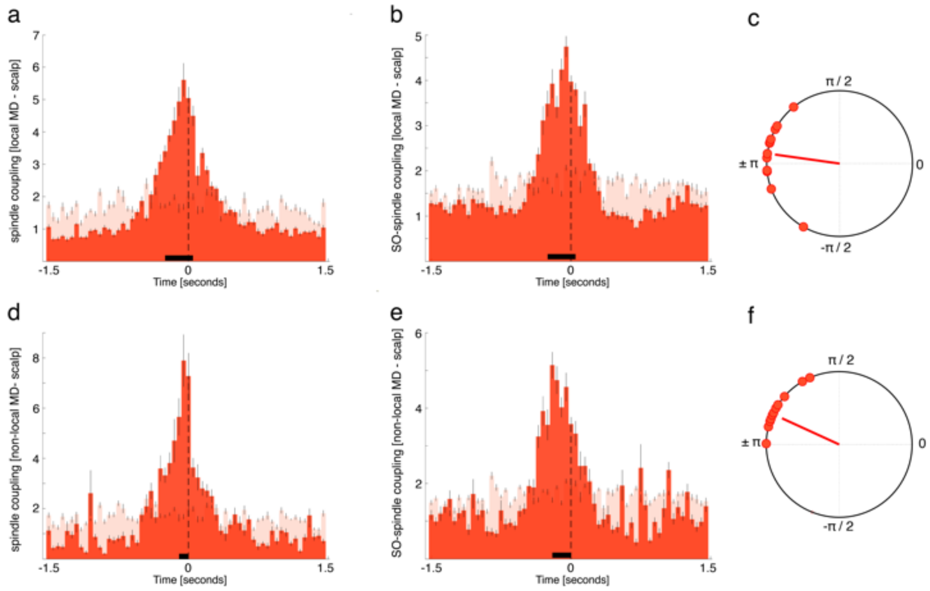

Supplementary Fig. 2 | Local and non-local MD spindles. Non-local MD spindles were defined as spindles identified in the MD whose peak (maximally negative amplitude) overlapped with ANT spindle peaks ± 50 ms (149.83 ± 35.69 MD spindles out of 2127.8 ± 238.2; 7.04%). All other spindles identified in the MD were defined as local MD spindles. The same temporal dynamics with regards to thalamo-cortical spindle coupling and SO-spindle coupling were found for local and non-local MD spindles. **(a)** Occurrence probabilities of local MD spindle peaks relative to neocortical spindle peaks (maximal negative amplitude, time = 0; dashed line; bin size = 50 ms), indicating that local MD spindles precede neocortical spindles (dependent-samples t-test, two-sided; positive cluster from -0.25 to 0 sec, $p < 0.0001$; corrected for multiple comparisons across time, time of peak: -0.05 sec). **(b)** Local MD spindles preferential emerged around MD SO down states (dependent-samples t-test, two-sided; -0.25 to 0.05 sec; $p < 0.0001$; corrected for multiple comparisons across time; peak: -0.05 sec). **(c)** Phases of the SO-spindle modulation derived from local MD events. Local MD spindles started specifically around the MD SO down states (mean coupling direction: −172.05° ± 7.60°; Rayleigh test, one-sided: $z = 9.59$, $p < 0.0001$). **(d)** Occurrence probabilities of non-local MD spindle peaks relative to neocortical spindle peaks, indicating that non-local MD spindles likewise precede neocortical spindles (dependent-samples t-test, two-sided; positive cluster from -0.1 to 0 sec, $p < 0.0001$; corrected for multiple comparisons across time; time of peak: -0.05 sec). **(e)** Non-local MD spindles also preferential emerged around MD SO down states (-0.2 to 0 sec; dependent-samples t-test, two-sided; $p = 0.017$; corrected for multiple comparisons across time; peak: -0.3 sec). **(f)** Phases of the SO-spindle modulation derived from non-local MD events. Non-local MD spindles started specifically around the MD SO down states (mean coupling direction: −155.38° ± 8.68°; Rayleigh test, one-sided: $z = 8.91$, $p < 0.0001$).

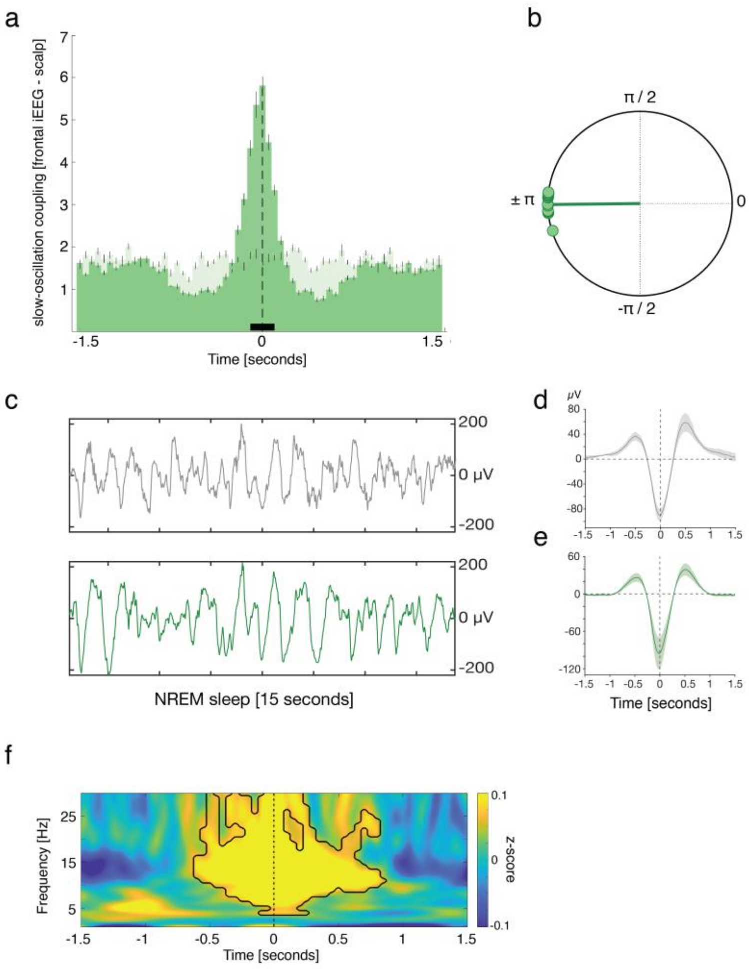

Supplementary Fig. 3 | SO coupling frontal iEEG – frontal scalp: To examine whether the skull, acting as a low pass filter, might have delayed the picked-up signal at the scalp level relative to thalamic recordings, an additional, independent data-set was analyzed. Specifically, SO coupling between frontal scalp electrodes (electrode FP1) and frontal intracranial contacts in three pre-surgical epilepsy patients (frontal iEEG contacts, N = 13) during NREM sleep was assessed. If the skull indeed delays the measured SO negative peak amplitude at the scalp level then a comparable delay of ~50 ms with regards to SOs should be observable in this data, were intracranial and scalp electrodes are in close distance. On average we detected 1450 ± 137.58 SOs in frontal intracranial contacts and 1245.7 ± 161.97 SOs in frontal scalp EEG recordings during NREM sleep, with a coupling rate of 30.15 ± 1.22 %. **(a)** Occurrence probabilities of intracranial detected SO down state peaks relative to scalp detected down state peaks (bin size = 50 ms), indicated that SOs in frontal intracranial and scalp recordings emerged synchronously (dependent-samples t-test, two-sided; positive cluster from -0.1 to 0.1 sec; $p < 0.0009$; corrected for multiple comparisons across time; time of peak = 0 sec). **(b)** We then determined the phase of intracranial SOs for all paired SO-events (i.e., all intracortical SOs within ± 750 ms of neocortical SOs) at the time of neocortical SO down state. We found a significant nonuniform distribution across contacts (Rayleigh test, one-sided: $z = 12.82$, $p < 0.0001$), with the phase of intracranial SOs being almost identical to the phase of scalp detected events (the phase of neocortical SO down states corresponds to ± π; mean coupling direction: −179.37 ± 1.84°). **(c)** Example of NREM sleep segment (15 sec), comprising SOs (top row: scalp recording; bottom row: intracranial). **(d)** Grand average EEG trace of neocortical SOs (mean ± SEM, negative peak, time 0). **(e)** Grand average iEEG trace of intracranial SOs (mean ± SEM, negative peak, time 0). **(f)** Time–frequency representation of neocortical SOs (locked to neocortical SO down states), contrasted against event-free segments. The contour lines indicate clusters (two-sided dependent-samples t-test; $p < 0.05$, corrected for multiple comparisons across time and frequency).

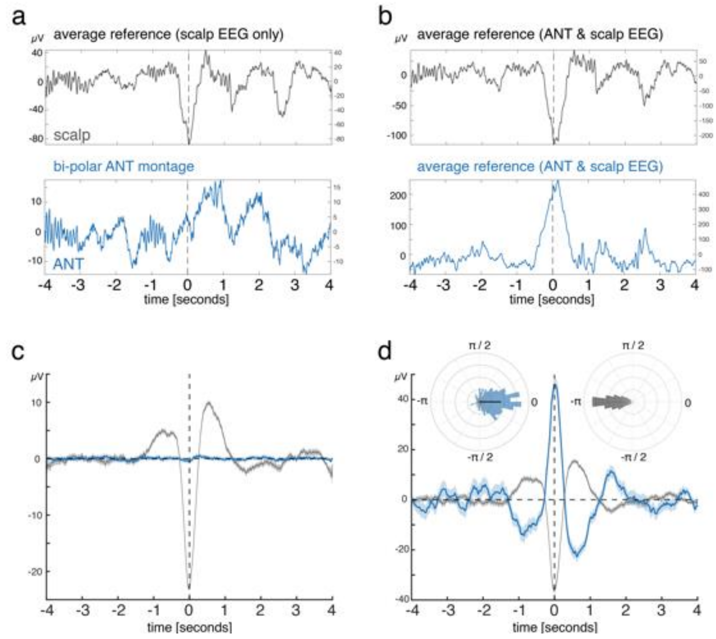

Supplementary Fig. 4| Estimating the potential influence of volume conducted SOs on the timing of thalamo-cortical SO coupling: While the bipolar montage represents the gold standard in order to resolve localized activity in intracranial recordings [37–39], volume conducted SOs might still intrude the measured signal. To accurately estimate the possible impact of volume conduction on our SO related results we ran additional control analyses on the data of a sample patient. First, we identified events where SOs are present in scalp EEG recordings (average referenced including scalp EEG electrodes only, as in the main analyses) but not in the bipolar referenced ANT recordings (for an example event see **(a)**; for ERPs of all identified events (scalp in grey, ANT in blue, see **(c)**). The same events were then isolated using a common average reference that included both scalp EEG and ANT recordings. The rationale of this procedure is to isolate SOs in the ANT that were exclusively a product of volume conduction and by that allowing to estimate potential technical delays in SO peaks between EEG and bipolar iEEG recordings. In sum, no such delay became apparent beyond 180 degree phase shift due to the polarity reversal of the ANT signal in the common average montage. **(b)** Shows the same example event as in (a) using a common average reference (including both scalp and intracranial recordings). **(d)** Illustrates all identified events used in (c) again using a common average reference (including both scalp (grey) and intracranial recordings (blue)), highlighting that SOs in scalp recordings and volume conducted SOs in the ANT, exhibiting a reversed polarity, peaked at the same time. The insets show in blue the phase of volume conducted SOs in ANT contacts at the time of neocortical SO downstate, illustrating that volume conducted SOs peaked at almost exactly the opposite phase as neocortical SOs (Rayleigh test, one-sided: $z = 282.71$, $p < 0.00001$; mean coupling direction: 1.59°). Cortical SO down-states using a common average reference (including iEEG as in b and d) peaked at 179.45° (Rayleigh test, one-sided: $z = 282.71$; $p < 0.00001$).

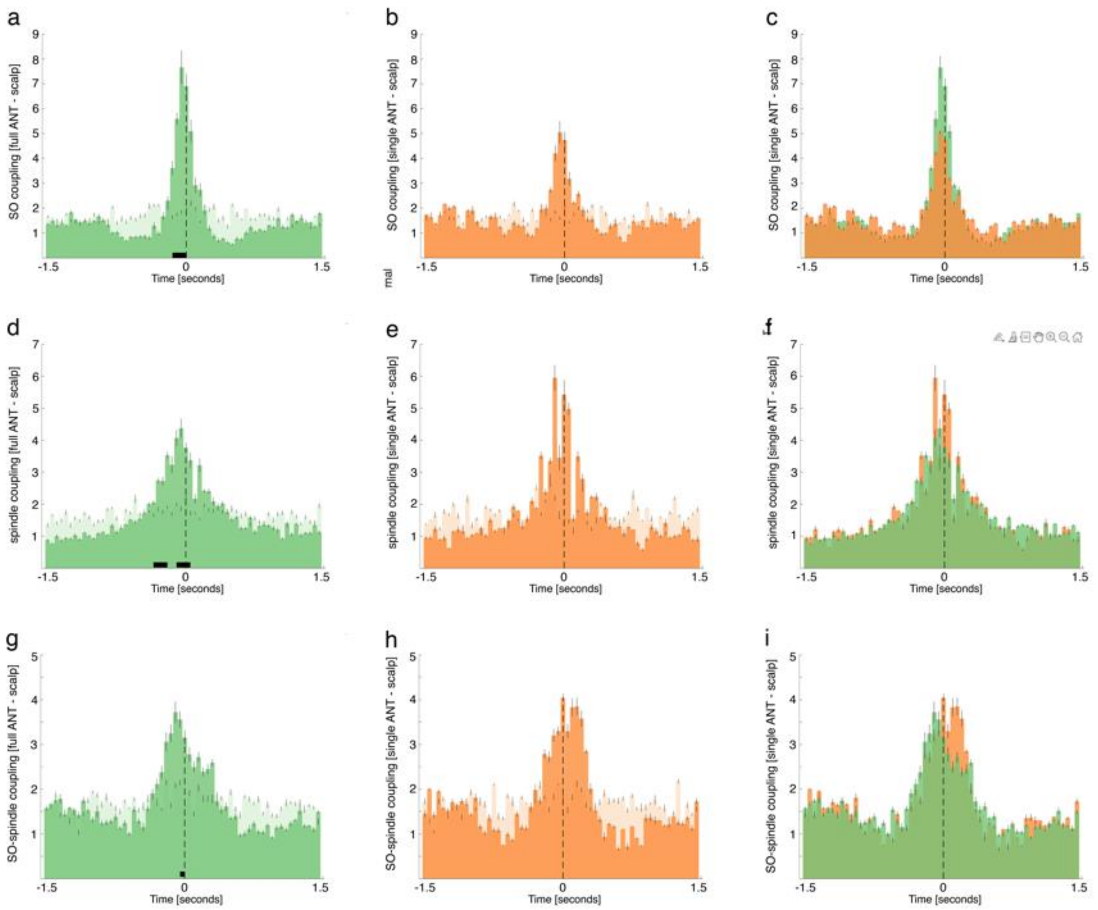

Supplementary Fig. 5 | ANT-scalp dynamics with regards to contact pairs were both contacts (n = 8; full ANT) or just one contact of a bipolar pair (n = 4, single ANT) were localized to the ANT: (a) Occurrence probabilities of full ANT SO down state peaks relative to scalp derived SO down state peaks (bin size = 50 ms), indicated that full ANT SOs preceded scalp derived SOs (dependent-samples t-test, two-sided; positive cluster from -0.15 to 0 sec; $p < 0.014$; corrected for multiple comparisons across time; time of peak = -0.05 sec). **(b)** Occurrence probabilities of single ANT SO down state peaks relative to scalp derived SO down state peaks (bin size = 50 ms). While single ANT SOs preceded descriptively scalp derived SOs, no significant differences were observable when tested against event-free occurrence probabilities (dependent-samples t-test, two-sided; $p > 0.1$; corrected for multiple comparisons across time). **(c)** No significant difference became apparent when directly comparing full ANT-scalp occurrence probabilities (green) and single ANT-scalp occurrence probabilities (orange) **(d)** Occurrence probabilities of full ANT spindle peaks relative to neocortical spindle peaks (maximal negative amplitude, time = 0; dashed line; bin size = 50 ms), indicating that full ANT spindles precede neocortical spindles (dependent-samples t-test, two-sided; positive clusters from -0.35 to -0.2 sec, $p < 0.001$ & -0.1 to 0.05 sec, $p < 0.001$; corrected for multiple comparisons across time; time of peak: -0.05 sec). **(e)** Again, single ANT sleep spindles, exhibited descriptively the same dynamics as full ANT spindle, but did not reach significance when tested against event-free occurrence probabilities (dependent-samples t-test, two-sided; $p > 0.1$, corrected for multiple comparisons across time). **(f)** No significant difference became apparent when directly comparing spindle related full ANT-scalp occurrence probabilities (green) and single ANT-scalp occurrence probabilities (orange). **(g)** Occurrence probabilities of full ANT spindle onsets with respect to full ANT SO down states (bin size = 50 ms), illustrating that spindles preferentially emerged around ANT SO down states (dependent-samples t-test, two-sided; -0.05 to 0 sec; $p = 0.048$, corrected for multiple comparisons across time). **(h)** The same SO-spindle related dynamic became apparent with regards to single ANT contact pairs, but again did not reach significance when tested against event-free occurrence probabilities (dependent-samples t-test, two-sided; $p > 0.1$, corrected for multiple comparisons across time). **(i)** Direct comparison of full and single ANT SO-spindle dynamics (green and orange, respectively) did not yield any significant difference (dependent-samples t-test, two-sided; $p > 0.1$, corrected for multiple comparisons across time).

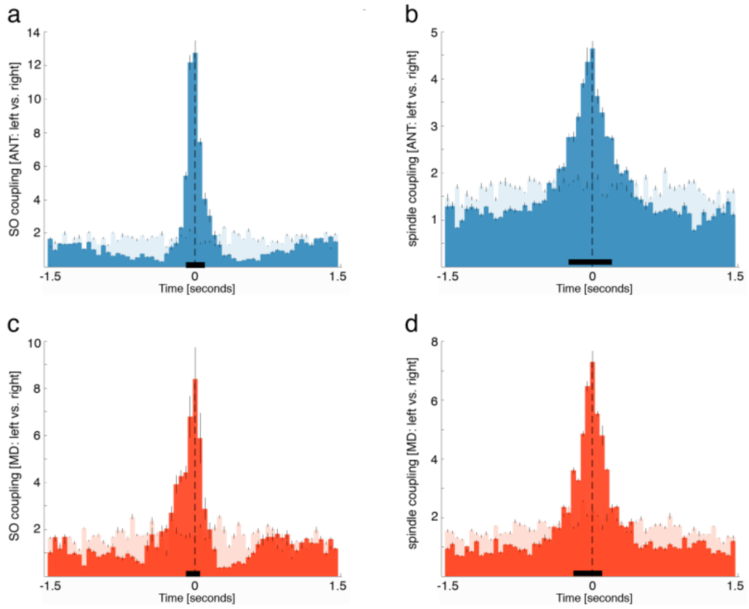

Supplementary Fig. 6 | Lateralization of coupling: (a) Occurrence probabilities of left ANT SO down state peaks relative to right ANT SO down state peaks (bin size = 50 ms), indicated that SOs in the left and right ANT emerged synchronously (dependent-samples t-test, two-sided; positive cluster from -0.1 to 0.1 sec; $p < 0.0001$; corrected for multiple comparisons across time; time of peak = 0 sec). **(b)** Occurrence probabilities for sleep spindles derived from the left ANT, relative to right ANT detected spindles, indicated that SOs in the left and right ANT emerged synchronously (dependent-samples t-test, two-sided; positive cluster from -0.25 to 0.25 sec; $p < 0.0001$; corrected for multiple comparisons across time; time of peak = 0 sec). **(c)** Occurrence probabilities of left MD SO down state peaks relative to right MD SO down state peaks, (dependent-samples t-test, two-sided; positive cluster from -0.15 to 0.05 sec; $p < 0.0001$; corrected for multiple comparisons across time; time of peak = 0 sec). **(d)** Occurrence probabilities for sleep spindles derived from the left MD, relative to right MD detected spindles (dependent-samples t-test, two-sided; positive cluster from -0.2 to 0.1 sec; $p < 0.0001$; corrected for multiple comparisons across time; time of peak = 0 sec).

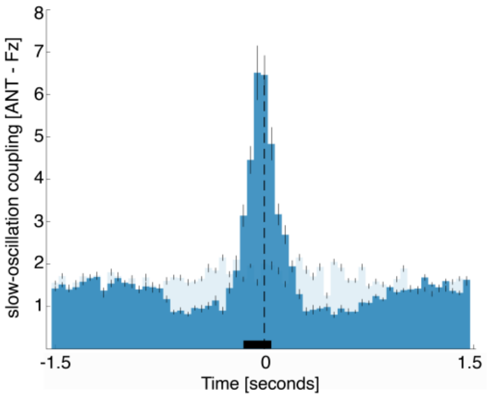

Supplementary Fig. 7 | SO coupling ANT-Fz: Occurrence probabilities of ANT SO down state peaks relative to SO down state peaks derived from scalp electrode Fz (time = 0; dashed line; bin size = 50 ms)), supporting the main finding that ANT SOs precede neocortical SOs. The solid black line indicates significant differences, resulting from comparison with SO-free control events (dependent-samples t-test, two-sided; positive cluster from -0.15 to 0.05 sec, $p < 0.005$; corrected for multiple comparisons across time; time of peak: -0.05 sec). Note that Fz was not used as generic seed electrode in the main analysis (Fig. 1a), as it was contaminated with epileptic activity in 3/8 patients.

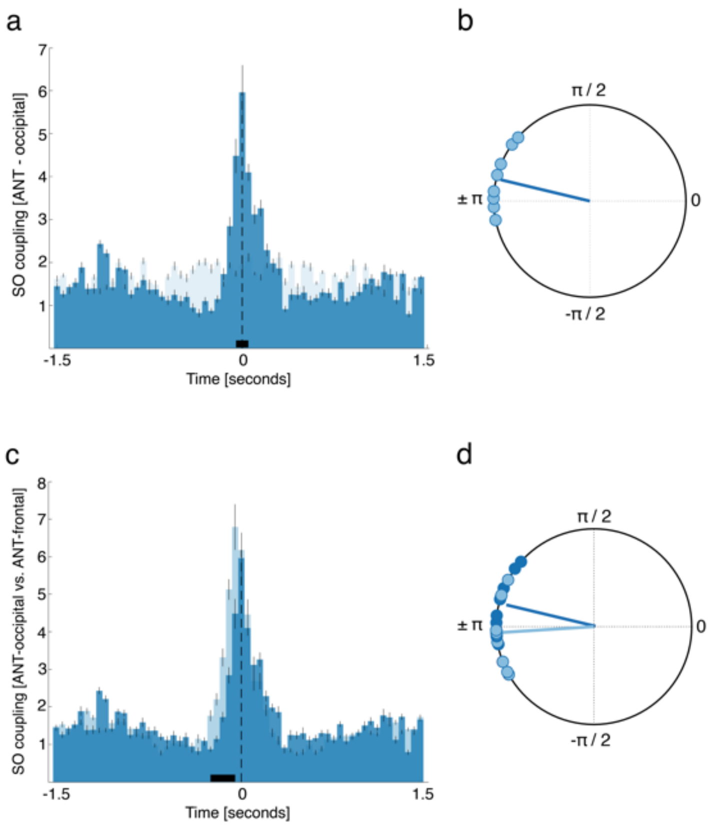

Supplementary Fig. 8 | SO coupling ANT-Occipital: To assess the specificity of ANT – frontal interaction with regards to SOs (Figure 2 a + b), the occurrence probabilities of ANT SOs relative to neocortical SOs derived from occipital electrodes (i.e., electrode O2) were evaluated. Due to missing / noisy occipital electrodes 2 patients (4 ANT contacts) had to be removed from this analysis. Occipital SOs that did not overlap with frontal detected SOs (± 750 ms, n = 1054.91 ± 118.38) were isolated, to ensure that the captured SOs are not mainly products of volume conduction. **(a)** Occurrence probabilities revealed, that SOs in the ANT did not precede, but peaked in parallel to occipital derived SOs (dependent-samples t-test, two-sided; positive cluster from -0.05 to 0.1 sec, $p = 0.01$; corrected for multiple comparisons across time; time of peak: 0.0 sec). **(b)** The phase of thalamic SOs for all paired SO-events at the time of occipital SO down state (± 750 ms, n = 176.12 ± 29.71; note that only 16.31 ± 1.88 % of ANT SOs were coupled to occipital derived SOs as compared to 33.74 ± 3.02 % in case of frontal SOs; dependent-samples t-test, two-sided; $t = -4.37$, $p = 0.0003$). A significant nonuniform distribution across contacts (Rayleigh test, one-sided: $z = 7.24$, $p < 0.001$) was detectable, with the phase of ANT SOs following their occipital counterparts (mean coupling direction: 166.56 ± 6.20°). **(c)** Direct comparison of occurrence probabilities for ANT-occipital and ANT-frontal SO interactions revealed a significant difference between the two distributions from -0.25 to -0.05 sec (dependent-samples t-test, two-sided; $p = 0.013$; corrected for multiple comparisons across time), indicating that ANT SOs preferentially emerged earlier with regards to frontal scalp SOs (light blue) as compared to occipital scalp SOs (dark blue). **(d)** Comparing the phase distributions for the coupling between thalamus and neocortex in relation to frontal and occipital SOs yielded a statistical trend in the direction that ANT SOs coupled to earlier phases of frontal SOs as compared to occipital SOs (Watson-Williams test, two-sided: $F = 4.12$, $p = 0.056$).

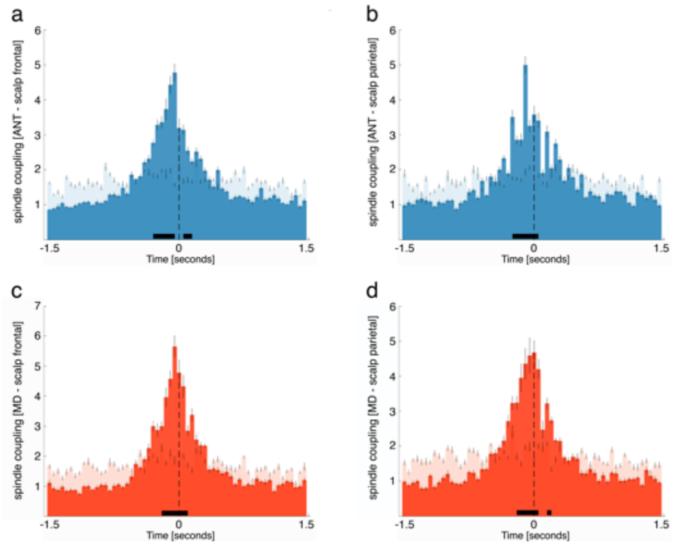

Supplementary Fig. 9 | Thalamic spindles coupled to frontal and parietal scalp spindles: (a + b) Occurrence probabilities of ANT spindle peaks relative to frontal **(a)** and parietal **(b)** neocortical spindle peaks (maximal negative amplitude, time = 0; bin size = 50 ms), indicating that ANT spindles precede neocortical spindles detected from both frontal and parietal scalp electrodes (dependent-samples t-tests, two-sided; frontal: first positive cluster from -0.3 to -0.05 sec, $p < 0.001$; second cluster from 0.05 to 0.15 sec, $p = 0.005$; time of peak: -0.05 sec; parietal: positive cluster from -0.25 to 0.05 s, $p < 0.001$; corrected for multiple comparisons across time). **(c + d)** Occurrence probabilities of MD spindle peaks relative to frontal **(c)** and parietal **(d)** neocortical spindle peaks (maximal negative amplitude, time = 0), indicating that MD spindles precede neocortical spindles detected from both frontal and parietal scalp electrodes (dependent-samples t-tests, two-sided; frontal: positive cluster from -0.2 to 0.1 sec, $p < 0.001$; time of peak: -0.05 sec; parietal: first significant positive cluster from -0.2 to 0.05 sec, $p < 0.001$; second positive cluster from 0.15 to 0.2 sec, $p = 0.01$; time of peak = 0; corrected for multiple comparisons across time).

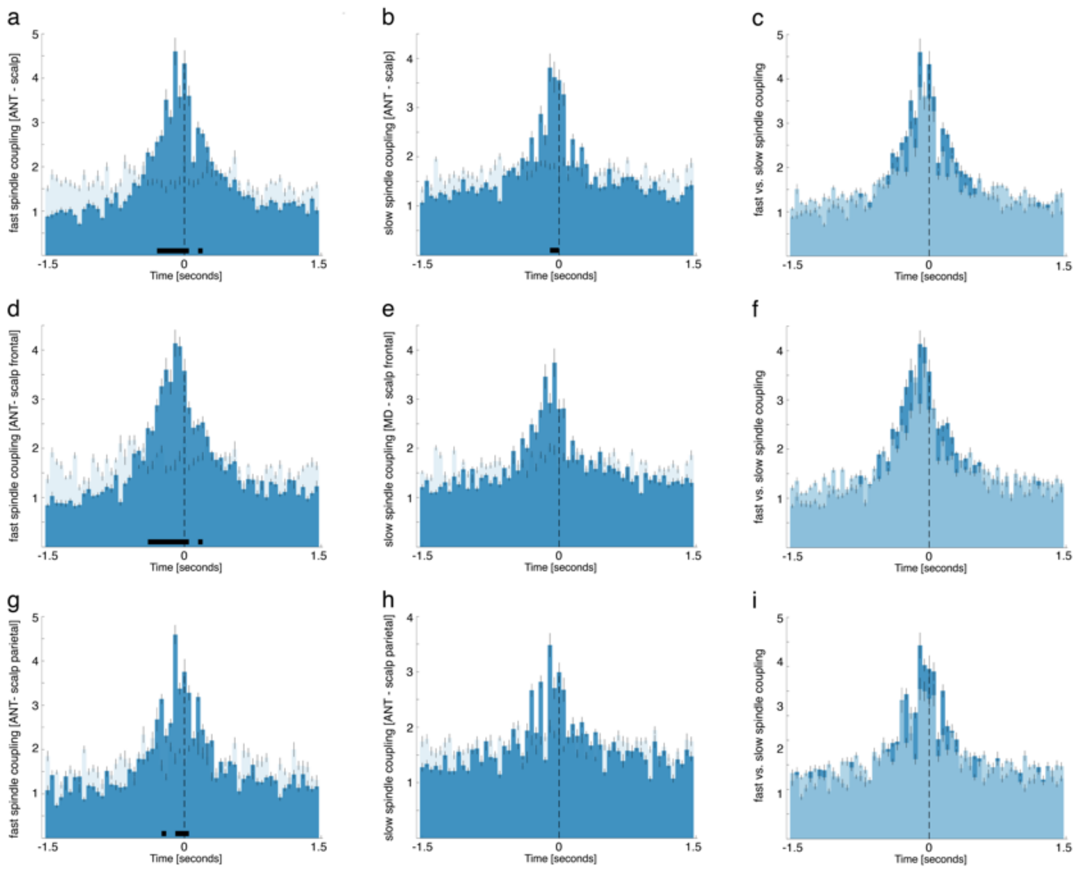

Supplementary Fig. 10 | ANT - cortical coupling for fast and slow spindles: Occurrence probabilities of ANT fast **(a)** and slow spindle **(b)** peaks relative to cortical spindles (same electrode sites as in the main analyses; for details see Supplementary table 2; maximal negative amplitude, time = 0; bin size = 50 ms). Both fast and slow ANT spindles significantly precede neocortical fast and slow spindles (dependent-samples t-tests, two-sided; fast spindles: first positive cluster from -0.3 to -0.1 sec, $p = 0.0007$; second cluster from 0.15 to 0.2 sec, $p = 0.015$; time of peak: -0.1 sec; slow spindles: positive cluster from -0.1 to 0.05 sec, $p = 0.001$; corrected for multiple comparisons across time). **(c)** the comparison of fast ANT-neocortical (dark blue) and slow ANT-neocortical SO interactions (light blue), did not yield significant differences (dependent-samples t-tests, two-sided: $p > 0.05$; corrected for multiple comparisons across time). **(d + e)** Occurrence probabilities for ANT fast and slow sleep spindles with regards to frontal neocortical fast and slow spindles. Fast ANT spindles significantly precede neocortical frontal fast spindles (dependent-samples t-tests, two-sided; first positive cluster from -0.4 to -0.05 sec, $p < 0.0001$; second cluster from 0.15 to 0.2 sec, $p = 0.008$; time of peak: -0.1 sec, corrected for multiple comparisons across time). In case of slow spindles, no significant differences were observable when tested against event-free occurrence probabilities (dependent-samples t-tests, two-sided: $p = 0.06$; corrected for multiple comparisons across time). **(f)** the comparison of frontal fast ANT-neocortical (dark blue) and slow ANT-neocortical SO interactions (light blue), did not yield significant differences (dependent-samples t-tests, two-sided: $p > 0.05$, corrected for multiple comparisons across time) **(g + h)** Occurrence probabilities for ANT fast and slow sleep spindles with regards to parietal neocortical fast and slow spindles. Fast ANT spindles significantly precede neocortical parietal fast spindles (dependent-samples t-tests, two-sided; first positive cluster from -0.25 to -0.2 sec, $p = 0.008$; second cluster from -0.1 to 0.05 sec, $p = 0.0002$; time of peak: -0.1 sec; corrected for multiple comparisons across time). In case of slow spindles, no significant differences were observable when tested against event-free occurrence probabilities (dependent-samples t-tests, two-sided; $p = 0.09$, corrected for multiple comparisons across time). **(i)** the comparison of parietal fast ANT-neocortical (dark blue) and slow ANT-neocortical SO interactions (light blue), did not yield significant differences (dependent-samples t-tests, two-sided: $p > 0.05$, corrected for multiple comparisons across time)).

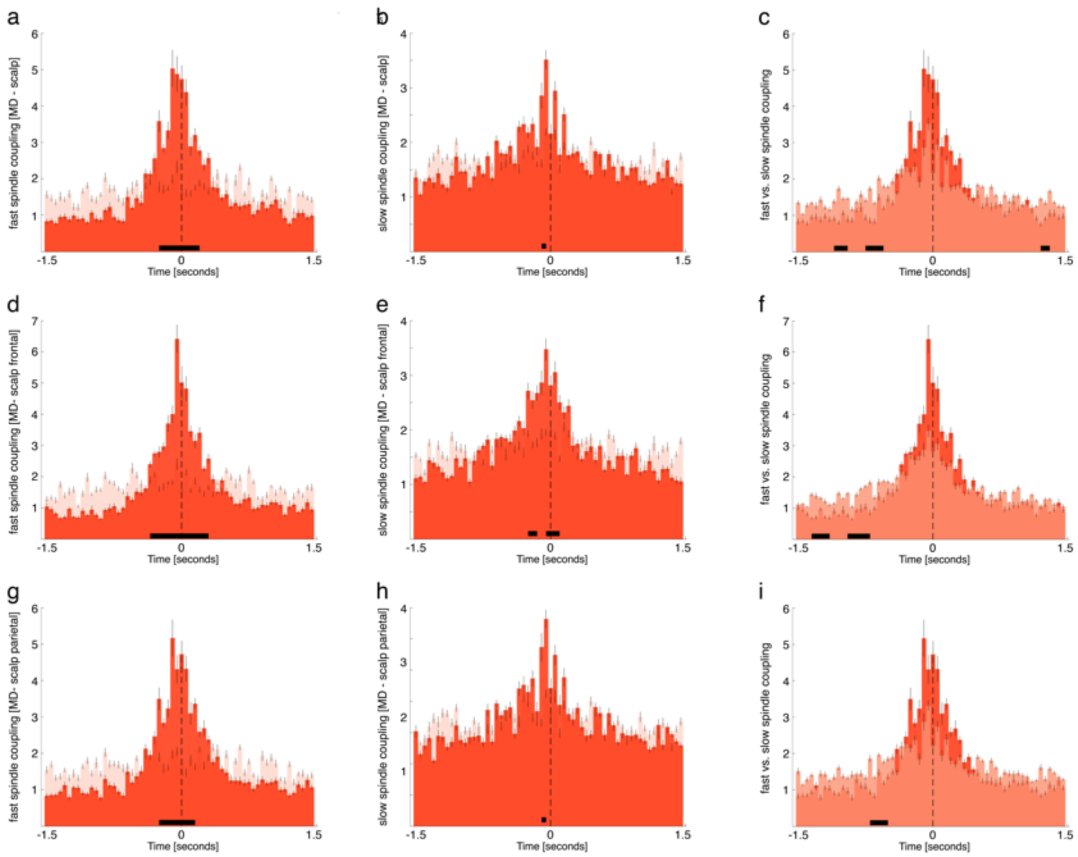

Supplementary Fig. 11 | MD - cortical coupling for fast and slow spindles: Occurrence probabilities of MD fast **(a)** and slow spindle **(b)** peaks relative to cortical spindles (same electrode sites as in the main analyses; for details see Supplementary table 2; maximal negative amplitude, time = 0; bin size = 50 ms). Both fast and slow MD spindles significantly precede neocortical fast and slow spindles (dependent-samples t-tests, two-sided; fast spindles: positive cluster from -0.3 to 0.2 sec, $p < 0.0001$; slow spindles: positive cluster from -0.1 to -0.05 sec, $p = 0.006$; corrected for multiple comparisons across time). **(c)** the comparison of fast MD-neocortical (dark red) and slow MD-neocortical SO interactions (light red), yielded multiple significant differences (dependent-samples t-tests, two-sided; negative cluster 1 from -0.75 to -0.5 sec, $p > 0.0001$; negative cluster 2 from -1.1 to -0.95 sec, $p = 0.002$; negative cluster 3 from 1.2 to 1.3 sec, $p = 0.004$; corrected for multiple comparisons across time). **(d + e)** Occurrence probabilities for MD fast and slow sleep spindles with regards to frontal neocortical fast and slow spindles. Both fast and slow MD spindles significantly precede neocortical frontal fast and slow spindles (dependent-samples t-tests, two-sided; fast spindles: positive cluster from -0.35 to 0.3 sec, $p < 0.0001$, time of peak: -0.05 sec; slow spindles: first positive cluster from -0.25 to -0.15 sec, $p = 0.002$; second positive cluster from -0.05 to 0.15 sec, $p < 0.0001$; time of peak: -0.05; corrected for multiple comparisons across time). **(f)** the comparison of fast frontal MD-neocortical (dark red) and slow MD-neocortical SO interactions (light red), yielded two negative clusters (dependent-samples t-tests, two-sided; negative cluster 1 from -0.95 to -0.7 sec, $p > 0.0001$; negative cluster 2 from -1.35 to -1.15 sec $p > 0.0001$; corrected for multiple comparisons across time). **(g + h)** Occurrence probabilities for MD fast and slow sleep spindles with regards to parietal neocortical fast and slow spindles. Both fast and slow MD spindles significantly precede neocortical parietal fast and slow spindles (dependent-samples t-tests, two-sided; fast spindles: positive cluster from -0.25 to 0.15 sec, $p < 0.0001$, time of peak: -0.1 sec; slow spindles: positive cluster from -0.1 to -0.05 sec, $p = 0.006$; time of peak: -0.05, corrected for multiple comparisons across time). **(i)** the comparison of fast parietal MD-neocortical (dark red) and slow MD-neocortical SO interactions (light red), one two negative clusters (dependent-samples t-tests, two-sided; negative cluster 1 from -0.7 to -0.5 sec, $p > 0.0001$; corrected for multiple comparisons across time).

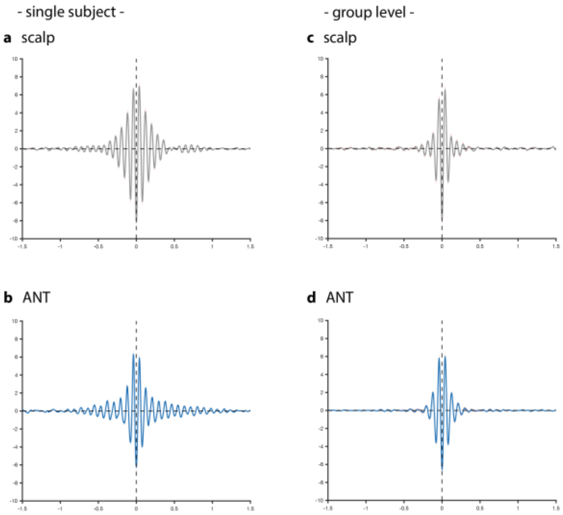

Supplementary Fig. 12 | ERPs sleep spindles single subject vs. group level: ERPs of **(a)** scalp level and **(b)** ANT detected sleep spindles for a sample participant. ERPs of **(c)** scalp level (1903.3 ± 211.5) and **(d)** ANT detected sleep spindles (N = 2203.8 ± 213.5) on the group level.
