# Supplementary material for: The human thalamus orchestrates neocortical oscillations during NREM sleep
Source: Nat Commun. 2022 Sep 5;13:5231. doi: 10.1038/s41467-022-32840-w (PMC9445182; doi:10.1038/s41467-022-32840-w)
Supplement: Supplementary file 3 — Reporting Summary [file 41467_2022_32840_MOESM3_ESM.pdf]

Corresponding author(s): Tobias Staudigl

Last updated by author(s): 04.08.2022

Reporting Summary

Nature Portfolio wishes to improve the reproducibility of the work that we publish. This form provides structure for consistency and transparency in reporting. For further information on Nature Portfolio policies, see our Editorial Policies and the Editorial Policy Checklist.

Statistics

For all statistical analyses, confirm that the following items are present in the figure legend, table legend, main text, or Methods section.

n/a	Confirmed	
☐	☒	The exact sample size (*n*) for each experimental group/condition, given as a discrete number and unit of measurement
☐	☒	A statement on whether measurements were taken from distinct samples or whether the same sample was measured repeatedly
☐	☒	The statistical test(s) used AND whether they are one- or two-sided<br>*Only common tests should be described solely by name; describe more complex techniques in the Methods section.*
☐	☒	A description of all covariates tested
☐	☒	A description of any assumptions or corrections, such as tests of normality and adjustment for multiple comparisons
☐	☒	A full description of the statistical parameters including central tendency (e.g. means) or other basic estimates (e.g. regression coefficient) AND variation (e.g. standard deviation) or associated estimates of uncertainty (e.g. confidence intervals)
☐	☒	For null hypothesis testing, the test statistic (e.g. F, t, r) with confidence intervals, effect sizes, degrees of freedom and P value noted<br>*Give P values as exact values whenever suitable.*
☒	☐	For Bayesian analysis, information on the choice of priors and Markov chain Monte Carlo settings
☒	☐	For hierarchical and complex designs, identification of the appropriate level for tests and full reporting of outcomes
☐	☒	Estimates of effect sizes (e.g. Cohen's *d*, Pearson's *r*), indicating how they were calculated

Our web collection on statistics for biologists contains articles on many of the points above.

Software and code

Policy information about availability of computer code

Data collection	Data were recorded using XLTEK Neuroworks software, version 9 (Natus Medical, San Carlos, California, US) and an XLTEK EMU128FS amplifier.
Data analysis	MATLAB 2020a ,Mathworks, https://uk.mathworks.com/;<br>Fieldtrip Toolbox v.09/01/2020, http://www.fieldtriptoolbox.org/;<br>CircStat Toolbox v.1, https://www.jstatsoft.org/article/view/v031i10;<br>LEAD-DBS v.1.6.3, https://github.com/netstim/leaddbs<br>SPM12, https://github.com/spm/spm12

For manuscripts utilizing custom algorithms or software that are central to the research but not yet described in published literature, software must be made available to editors and reviewers. We strongly encourage code deposition in a community repository (e.g. GitHub). See the Nature Portfolio guidelines for submitting code & software for further information.

Data

Policy information about availability of data

All manuscripts must include a data availability statement. This statement should provide the following information, where applicable:

- Accession codes, unique identifiers, or web links for publicly available datasets
- A description of any restrictions on data availability
- For clinical datasets or third party data, please ensure that the statement adheres to our policy

Source data are provided with this paper. The raw data are protected and are not openly available due to data privacy laws, though (subject to these privacy laws) the data are available upon reasonable request.

Field-specific reporting

Please select the one below that is the best fit for your research. If you are not sure, read the appropriate sections before making your selection.

☒ Life sciences ☐ Behavioural & social sciences ☐ Ecological, evolutionary & environmental sciences

For a reference copy of the document with all sections, see nature.com/documents/nr-reporting-summary-flat.pdf

Life sciences study design

All studies must disclose on these points even when the disclosure is negative.

Sample size	The main population comprised 8 patients (11 full-night recordings). This sample size was solely driven by the supply of patients (an incredibly rare sample). Addtionally, 3 patients were included in an indpendent supplementary anaysis in response to a reviewer's request.
Data exclusions	The data of 5 patients were discarded prior to analyses due to excessive epileptic activity. Artifactual trials and channels were removed from the remaining data to ensure that the effects we observed were not driven by noise.
Replication	We did not replicate the present findings in an independent cohort, due to the rarity of the recordings.
Randomization	Not applicable - Randomization was not relevant due to the single group, within-subject design of the study.
Blinding	Not applicable - Blinding was not relevant, as a single group, within-subject design was used.

Reporting for specific materials, systems and methods

We require information from authors about some types of materials, experimental systems and methods used in many studies. Here, indicate whether each material, system or method listed is relevant to your study. If you are not sure if a list item applies to your research, read the appropriate section before selecting a response.

Materials & experimental systems

n/a	Involved in the study	
☒	☐	Antibodies
☒	☐	Eukaryotic cell lines
☒	☐	Palaeontology and archaeology
☒	☐	Animals and other organisms
☐	☒	Human research participants
☒	☐	Clinical data
☒	☐	Dual use research of concern

Methods

n/a	Involved in the study	
☒	☐	ChIP-seq
☒	☐	Flow cytometry
☒	☐	MRI-based neuroimaging

Human research participants

Policy information about studies involving human research participants

Population characteristics	8 patients (mean age: 38.65 ± 3.59; 5 female) took part. All patients were adults with drug-resistant epilepsy who received neurostimulation with ANT-DBS. Please note that more detailed information on the diagnosis is irrelevant for the purpose of the paper and may potentially violate data-protection agreements.
Recruitment	Patient recruitment involved asking those undergoing the relevant surgery to take part following the implanation of the DBS electrodes (Epilepsy Center, Department of Neurology, Ludwig-Maximilian Universität, Germany).
Ethics oversight	The measurements were approved by the Ethics Commission of the Medical Faculty of the Ludwig-Maximilian Universität

Ethics oversight	München.

Note that full information on the approval of the study protocol must also be provided in the manuscript.
